# Assessing social protection influence on health status in the European Union

**DOI:** 10.3389/fpubh.2024.1287608

**Published:** 2024-03-11

**Authors:** Claudiu George Bocean, Anca Antoaneta Vărzaru

**Affiliations:** ^1^Department of Management, Marketing and Business Administration, Faculty of Economics and Business Administration, University of Craiova, Craiova, Romania; ^2^Department of Economics, Accounting and International Business, Faculty of Economics and Business Administration, University of Craiova, Craiova, Romania

**Keywords:** healthcare, health status, healthcare expenditure, social protection, social protection expenditure

## Abstract

**Introduction:**

Health status and access to healthcare services are crucial factors that directly impact the well-being of individuals and societies. In the European Union (EU), social protection measures are significant in supporting citizens’ health and providing access to healthcare resources.

**Methods:**

This study investigates the relationship between social protection and health status in EU countries. We collected data from Eurostat on the EU member states’ health status, healthcare expenditure, and social protection expenditure. The paper used structural equation modeling (SEM) and cluster analysis to analyze the complex interplay among these variables.

**Results:**

Findings revealed a strong positive correlation between EU countries’ social protection expenditure and healthcare status. Higher social protection spending was associated with improved access to healthcare services and facilities. Moreover, the analysis showed that countries with higher social protection expenditure tended to exhibit better overall health status indicators among their populations.

**Discussion:**

The results suggest that adequate social protection expenditure positively influences health status in the European Union. By investing in robust social protection programs, governments can enhance citizens’ access to healthcare services and resources, ultimately leading to improved health outcomes. These findings underscore the importance of prioritizing social protection policies to address health disparities and promote public health in the EU.

## Introduction

Health status is a fundamental indicator of a society’s well-being and development. In the European Union (EU) context, promoting and maintaining good health among its citizens is a top priority for ensuring a prosperous and equitable future. Social protection measures have long been recognized as crucial in safeguarding individuals and communities from various risks, including health-related risks. These protective mechanisms, from health insurance schemes to social assistance programs, are pivotal in supporting vulnerable populations and enhancing access to essential healthcare services.

The current state of healthcare and social protection in the European Union (EU) is heavily influenced by the overarching goal of promoting the well-being and development of its citizens. With a focus on ensuring prosperous and equitable futures, EU member states prioritize measures that support good health and provide social protection against various risks, including those related to health. However, these systems face significant challenges, particularly in light of factors such as population aging, financial crises, and the COVID-19 pandemic. These pressures highlight the need for modernization to improve efficiency in resource allocation, both financial and human, within healthcare systems.

Improving the efficiency of financial and human resource allocation to healthcare systems within the European Union represents a priority for European social protection and healthcare strategies. The health of the European population is monitored through indicators such as population health improvement concerning the quality of healthcare services, attracting and retaining specialists in the medical system, or enhancing the quality of life in connection with new treatments and medical protocols in the field (1). The fragmentation of the healthcare system and inadequate social protection, as observed in the American system, may generate challenges to the population’s health, which can rapidly deteriorate during pandemics, as seen in 2020 during the COVID-19 pandemic (2). The relationship between social protection and health status has become a subject of increasing interest among policymakers, researchers, and public health experts. Understanding how social protection influences health outcomes in the EU is vital for developing effective and targeted policies to address health disparities and foster better health for all citizens.

Recognizing the crucial link between social protection and health outcomes, policymakers, researchers, and public health experts are increasingly interested in understanding how social protection initiatives impact the health of EU citizens. This understanding is essential for the development of targeted policies aimed at addressing health disparities and promoting better health outcomes for all.

The research gap that the study aims to address within the broader context of healthcare and social protection in the European Union is the need for a comprehensive understanding of the intricate connections between social protection initiatives and health indicators. While it is widely recognized that social protection plays a vital role in promoting better health outcomes and mitigating health disparities, there is still a lack of in-depth analysis regarding the specific mechanisms through which these programs contribute to improved health status among EU residents.

The paper seeks to provide evidence-based insights into the significant role of social protection in shaping health outcomes within the EU context to address this gap. We aim to fill this gap by employing a comprehensive approach that combines robust data analysis with relevant theoretical frameworks. Through empirical study, we aim to identify and elucidate the pathways through which social protection measures influence various health indicators, such as access to healthcare services, health behaviors, and health outcomes. Furthermore, the paper aims to make several significant contributions to the existing body of knowledge on healthcare and social protection within the European Union.

The COVID-19 pandemic has highlighted the critical importance of robust healthcare systems and comprehensive social protection measures in safeguarding the well-being of EU citizens. The pandemic has exposed vulnerabilities in healthcare infrastructures and exacerbated existing health disparities (3), underscoring the urgent need for effective policies that address these shortcomings (4). This study contributes to ongoing efforts to strengthen healthcare and social protection systems within the European Union, providing valuable insights that are particularly relevant in light of recent events like the COVID-19 pandemic.

The paper’s structure is as follows: after reviewing the existing literature, the paper exposes the methodological framework. The results of this study are expected to offer evidence-based insights into the significant role of social protection in shaping health outcomes in the EU. The discussions will contribute to a deeper understanding of the complex dynamics between social protection and health status. The conclusions encapsulate the findings of the paper.

## Literature review

The World Health Organization (WHO) defines public health as “the organized efforts of society to promote, protect, improve, and restore the health of individuals, specified groups, or the entire population” (5). Essentially, public health is a comprehensive and integrated approach that aims to improve the health status of the entire population by promoting a healthy environment, preventing diseases, and promptly addressing existing health issues.

The efficiency of healthcare services has been the subject of extensive debates and concerns in health economics in recent years (6). This action involves providing accessible and patient-centered medical care by allocating limited healthcare resources and rejecting certain potentially beneficial programs or treatments for specific individuals. It is a suitable practice to ensure a rational, equitable, and cost-effective allocation of healthcare resources (6). Rising healthcare costs and demands have placed considerable pressure on health authorities to develop effective strategies for resource allocation. Efficient healthcare services require identifying mechanisms for cost-effectively allocating limited resources and ensuring maximum benefits for patient populations (6–9).

The economic perspective regarding healthcare systems becomes increasingly important, considering the escalating costs and growing burden of diseases (10–12). In this context, optimizing these systems becomes crucial to ensure optimal utilization of limited resources and providing quality services at sustainable costs. Economic evaluation plays a vital role in the decision-making process for healthcare organizations and practitioners, providing valuable information regarding the impact and effectiveness of various policies and programs (6).

Despite the importance of these economic evaluations, a decline in healthcare resources remains a significant challenge, particularly in low-income countries or during periods of economic recession and public spending cuts (11, 13–16). The COVID-19 pandemic has further amplified the pressure on limited healthcare resources, presenting health systems with new challenges. In these challenging circumstances, adopting efficient policies and measures based on rigorous economic evaluations becomes even more critical to ensure effective resource management and adequate population protection.

Various authors (17–20) have addressed structuring public health financial allocations in studies conducted within European countries. In Eastern Europe, Cacace (19) observes a need to improve financial allocations for health systems, given the growing receipts for general social insurance. Abor and Abor (21) and Béland et al. (22) recommend budgetary rebalancing measures, increased collection rates, and efficient utilization of allocated financial resources in the healthcare system. Kwon and Kim (23) believe that enhancing the resilience and sustainability of the healthcare system is necessary through strategies targeting disease prevention and control, health monitoring, and efficient population information through the digitization of administrative and medical systems.

Healthcare allocations differ based on economic performance, contributing to poor performance when the healthcare system is underfunded (24–26). A proactive policy in healthcare funding is necessary, as a healthy population leads to more robust economic growth and requires fewer social protection services.

At the same time, research in health economics continues to play a vital role in addressing issues of accessibility and equity in healthcare delivery. Academic works in this field explore ways to streamline expenses, identify more cost-effective medical practices, and prioritize medical services for vulnerable groups (10–12). Research can contribute to shaping appropriate policies and strategies to address healthcare system challenges while ensuring universal access to essential medical services.

The European Union (EU) places significant attention on the implications of its policies on public health and social security to ensure equal access to high-quality and affordable medical and social services. The EU supports member states in achieving common objectives and promotes cooperation among countries to address common challenges (27). In health policies, the EU focuses on strategic objectives, including promoting good health, protecting citizens from cross-border health threats, supporting dynamic healthcare systems, and facilitating better access to medical services for EU citizens. However, various challenges remain, such as addressing the specific health needs of an aging population, adapting to demographic changes, reducing avoidable diseases, and tackling emerging health issues such as antimicrobial resistance.

Social protection services, including social assistance, are considered by Yokobori et al. (28) to play an essential role in improving access to healthcare services among vulnerable populations. Multiple systematic analyses have demonstrated the positive impact of social insurance on health. For example, social protection and social insurance provision have been associated with improved maternal and child health service utilization (28–30). Spaan et al. (31) identify positive effects on health resulting from improvements in the health insurance system. However, Acharya et al. (32) found that improving the health insurance system did not significantly improve health for vulnerable groups, only benefiting the overall population. Sustainable and well-managed social programs can improve individual health and contribute to overall public health by creating a favorable health development and maintenance environment. These findings have significant practical and managerial implications (28). Integrating public health perspectives into developing and implementing social policies can create more comprehensive and targeted approaches to improving population health (27).

Based on previous research findings, we have formulated the first hypothesis of the research: Hypothesis H1. Social protection expenditure positively influences health status, alongside healthcare expenditure, within the European Union countries.

Numerous studies have highlighted the positive association between social protection expenditure and health outcomes (24–29). Social protection programs, such as universal healthcare coverage, income support, and social welfare initiatives, are designed to mitigate socio-economic disparities and provide individuals with access to essential healthcare services and resources (33–37). Ensuring equitable access to healthcare and addressing social determinants of health, social protection expenditure is expected to impact health status positively, leading to improved overall well-being and reduced morbidity and mortality rates (28).

Moreover, the synergy between social protection expenditure and healthcare expenditure is essential to achieve optimal health outcomes. While healthcare expenditure directly contributes to the provision of medical services and treatments, social protection expenditure complements these efforts by addressing broader social determinants of health, such as poverty, education, and housing (38–40). By investing in both healthcare infrastructure and social protection programs, countries can create a supportive environment conducive to better health outcomes for their populations (19, 20). The research gap lies in the need for a comprehensive understanding of the intricate connections between social protection initiatives and health indicators.

**Figure 1:**
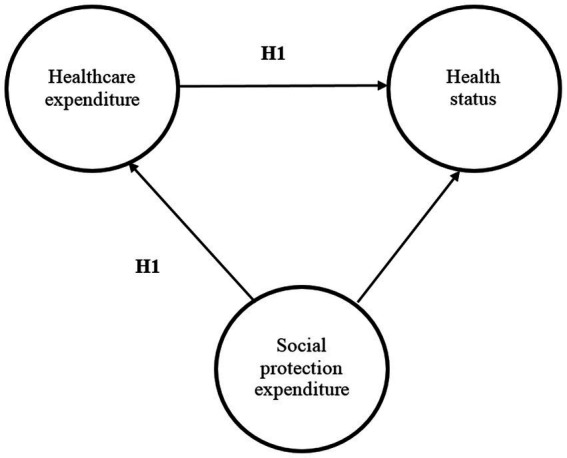
The research theoretical model. Source: authors’ design based on literature review.

Figure 1 presents the theoretical model of the research. The proposed framework elucidates the interplay between social protection policies, healthcare spending, and population health outcomes, highlighting the complex relationships and pathways through which these factors interact. At its core, the conceptual framework is grounded in the recognition of the pivotal role that social protection mechanisms play in promoting health and well-being. Social protection expenditure encompasses a range of policies and programs aimed at addressing socio-economic inequalities, ensuring access to essential services, and mitigating the impact of adverse social determinants on health. In tandem with social protection expenditure, healthcare expenditure represents a critical determinant of population health outcomes. Healthcare expenditure encompasses the financial resources allocated toward healthcare infrastructure, medical services, and public health initiatives. Healthcare systems and services investments aim to improve access to quality healthcare, enhance preventive measures, and address the burden of disease, ultimately contributing to better health outcomes among the population.

The conceptual framework postulates that social protection expenditure and healthcare expenditure are complementary and synergistic in their effects on health status. Social protection policies create an enabling environment conducive to better health outcomes. Healthcare expenditure, on the other hand, directly impacts access to medical services, treatments, and preventive care, thereby influencing individual and population health.

Within the European Union, the healthcare system is closely correlated with the social protection system to ensure access to healthcare services for vulnerable populations (41). This interconnectedness is fundamental in addressing health inequalities and ensuring that no person is left behind due to financial resources or other social constraints. Numerous academic papers emphasize the importance of extensive social protection services, including social assistance, during the COVID-19 pandemic (3, 4, 42). In this pandemic, vulnerable populations have been more exposed to health and social risks, and social protection services have proved essential in providing the necessary support to overcome difficulties and challenges.

The European Union has implemented collaborative strategies and programs that respond to common challenges in social protection and health (43). The healthcare domain can play an essential role in increasing the active working population and, therefore, can promote social inclusion and combat poverty (44). Various authors bring essential aspects of the link between social protection and public health to the forefront, emphasizing the positive impact of sustained well-being through significant social protection measures on the population’s overall health status (45–47). Researchers have identified that expanding and strengthening social protection systems can benefit public health, with countries having robust social protection programs showing better outcomes in this area (47–50).

McCartney et al. (50) and Ullah and Harrigan (47) have highlighted several relevant aspects regarding the influence of social protection on public health. These studies have shown that well-designed social policies, such as health insurance, social assistance, pensions, or support for people with disabilities, positively impact population health. Social protection can reduce health inequalities and improve health indicators for the entire population by ensuring access to quality medical services and adequate financial support.

Academic literature (1, 6) signals financial allocations for healthcare and social protection services imbalances. Social protection must include easy and free access to healthcare services for vulnerable populations exposed to poverty and aging to promote equitable healthcare access. Better health for vulnerable categories and prolonged active life improve the quality of life and reduce social protection expenses.

Structural reforms in social protection systems and investments in healthcare must go hand in hand to achieve public policy objectives and ensure universal access to quality medical care. Health investments are crucial to ensuring a healthy and equitable society within the European Union. A more efficient healthcare system can be achieved through responsible resource management and the implementation of appropriate reforms capable of meeting present challenges and addressing the health needs of EU citizens (43).

Based on previous research findings, we have formulated the second hypothesis of the research: Hypothesis H2. EU countries can be grouped into homogeneous clusters based on health status, healthcare expenditure, and social protection expenditure. This hypothesis is rooted in the recognition of inherent variations among EU member states regarding their healthcare systems, social protection policies, and health outcomes.

Numerous empirical studies have demonstrated the existence of distinct patterns and similarities among countries in terms of health status, healthcare expenditure, and social protection expenditure (17–19, 24–26). These variations can be attributed to diverse socio-economic, political, and cultural factors that shape the healthcare landscape and social welfare policies across different EU nations. Furthermore, understanding the heterogeneity among EU countries is crucial for informing evidence-based policymaking and facilitating knowledge exchange and collaboration among member states. Homogeneous clusters provide insights into best practices implemented by countries with comparable profiles, thereby promoting mutual learning and fostering innovation in healthcare and social protection policies.

Efforts to improve healthcare and social protection systems must be integrated and coherent to ensure the health and well-being of all citizens. In this regard, collaboration among decision-makers, healthcare and social assistance experts, and representatives from civil society is essential to identify the most efficient and sustainable solutions (26). Social protection and access to healthcare are interdependent and play a crucial role in ensuring an equitable and inclusive healthcare system for all citizens.

## Materials and methods

This research investigates the relationship between social protection and health status in European Union countries. To achieve this objective, we collected data from Eurostat on health status, healthcare expenditures, medical resources, and social protection expenditures in 2020 for EU member states. Table 1 presents the research variables. Eurostat provides comprehensive and reliable data on various socio-economic indicators for EU member states. The indicators selected align closely with the research objectives, which aim to examine the relationship between social protection, healthcare expenditures, and health outcomes within the European Union. By using data from Eurostat and other official sources, we can effectively address the research objectives and generate evidence-based insights.

**Table 1:** Selected variables.

Variable	Dataset	Measure
HS1	Share of people with good or very good perceived health by sex	Percentage
HS2	Healthy life years at age 65 by sex	Year
HE1	Total healthcare expenditure	Euro per inhabitant
HE2	Total healthcare expenditure	Percentage of gross domestic product (GDP)
SPE1	Total expenditure	Euro per inhabitant
SPE2	Social protection benefits	Net social protection as a percentage of GDP

Source: authors’ design based on collected data from Eurostat.

This study used Structural Equation Modeling (SEM) and cluster analysis to examine the complex interaction between these variables. SEM allows examining social protection expenditures’ direct and indirect effects on health status while controlling for potential confounding factors. This methodological approach is suitable for investigating the complex relationships and interdependencies between social protection and health status in the context of diverse countries in the European Union. Furthermore, SEM offers several distinct advantages over alternative methods. Traditional regression analysis, for instance, may struggle to capture the complex interdependencies inherent in social protection systems and their impact on health outcomes (51). Additionally, while econometric techniques such as panel data analysis can account for temporal dynamics, they may not adequately capture the intricate causal pathways and feedback loops characteristic of social protection and health interactions. SEM, on the other hand, provides a flexible and robust framework for modeling such complex relationships, making it an ideal choice for this study’s analytical needs (52).

Cluster analysis will enable the identification of groups of countries with similar characteristics regarding social protection, healthcare expenditures, and health status. This analysis will provide a deeper insight into the existing variations among EU member states and help us understand how specific factors can influence the relationship between social protection and population health.

In conjunction with SEM, cluster analysis is employed further to enrich the understanding of the heterogeneity among EU member states in terms of social protection, healthcare expenditures, and health status. By identifying distinct clusters of countries with similar characteristics, this analysis facilitates the identification of patterns that may not be immediately apparent through traditional statistical methods. Moreover, cluster analysis complements SEM by providing a visual and intuitive representation of the underlying structures within the data, thus enhancing the interpretability and applicability of the findings.

The integration of SEM and cluster analysis within the methodological framework of this study allows for a comprehensive and nuanced exploration of the complex relationships between social protection and health status in the European Union. By leveraging the strengths of these analytical techniques, the paper aims to uncover novel insights that can inform evidence-based policymaking and contribute to the enhancement of public health and social welfare across the region.

## Results

**Figure 2:**
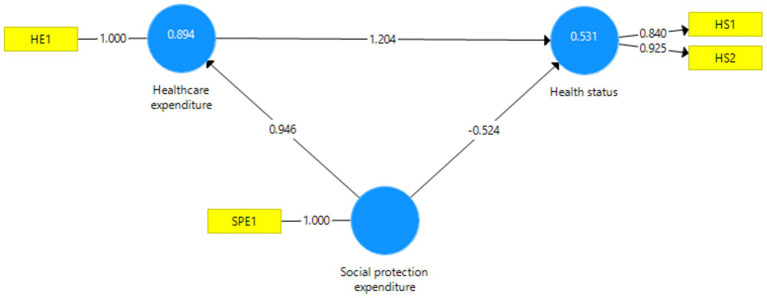
Empirical model. Source: authors’ design using SmartPLS v.3.0 (53).

Investigating hypothesis H1 involved using Partial Least Squares Structural Equation Modeling. The software utilized for hypothesis testing was SmartPLS v3.0. The theoretical model was tested, with each latent variable having two observable variables: health status (HS1 and HS2), healthcare expenditure (HE1 and HE2), and social protection expenditure (SPE1 and SPE2). The obtained model showed SRMR 0.148 and NFI 0.357, as well as high reliability. The descriptive statistics are presented in Table 2. To enhance model reliability and validity, we removed the observable variables reported to GDP (HE1 and SPE2), which affected the relationship between latent variables, as recommended by Hair et al. (3). The resulting valid model is illustrated in Figure 2.

**Table 2:** Descriptive statistics.

	*N*	Minimum	Maximum	Mean	Std. deviation	Skewness	Kurtosis
HS1	27	44.30	83.70	68.1296	9.41140	−0.868	0.537
HS2	27	4.40	15.90	9.0444	2.73163	0.252	0.041
HE1	27	712.57	5875.34	2858.4474	1790.67771	0.423	−1.470
HE2	27	5.77	12.82	9.1744	1.94804	0.019	−1.089
SPE1	27	1661.23	24823.13	8310.2726	5922.09617	0.967	0.475
SPE2	27	14.16	33.09	23.1756	4.91408	0.065	−0.935

Source: authors’ design based on collected data from Eurostat.

The model exhibits good fit indices (SRMR 0.075 and NFI 0.904). The reliability of the latent variable, health status, characterized by two observable variables, is excellent (Cronbach’s Alpha 0.727; Composite Reliability 0.877; Average Variance Extracted 0.781). The model’s discriminant validity is also excellent, as depicted in Table 3 using the Fornell–Larcker criterion (4).

**Table 3:** Discriminant validity.

	Health status	Healthcare expenditure	Social protection expenditure
Health status	0.884		
Healthcare expenditure	0.709	1	
Social protection expenditure	0.615	0.946	1

Source: authors’ design using SmartPLS v.3.0.

Using a bootstrapping procedure, we obtained path coefficients within the model, illustrating direct and indirect relationships between latent variables. Table 4 presents the total and indirect effects recorded between latent variables.

**Table 4:** Specific indirect and total effects.

	Original sample	Sample mean	Standard deviation	*T* statistics	*p* values
Social protection expenditure - > Healthcare expenditure - > Health status	1.139	1.179	0.467	2.437	0.015
Healthcare expenditure - > Health status	1.204	1.237	0.48	2.508	0.012
Social protection expenditure - > Health status	0.615	0.632	0.076	8.077	0.000
Social protection expenditure - > Healthcare expenditure	0.946	0.953	0.019	49.483	0.000

Source: authors’ design using SmartPLS v.3.0.

The relationships from Table 4 confirm the validity of hypothesis H1. Social protection expenditures significantly positively influence health status, but healthcare expenditure mediated this influence. Social protection is typically associated with substantial vulnerable groups with poor health status (8–10), which results in a negative direct relationship between social protection expenditure and health status. Social protection should be directed towards health-related expenditures to involve more individuals in the active working population.

**Figure 3:**
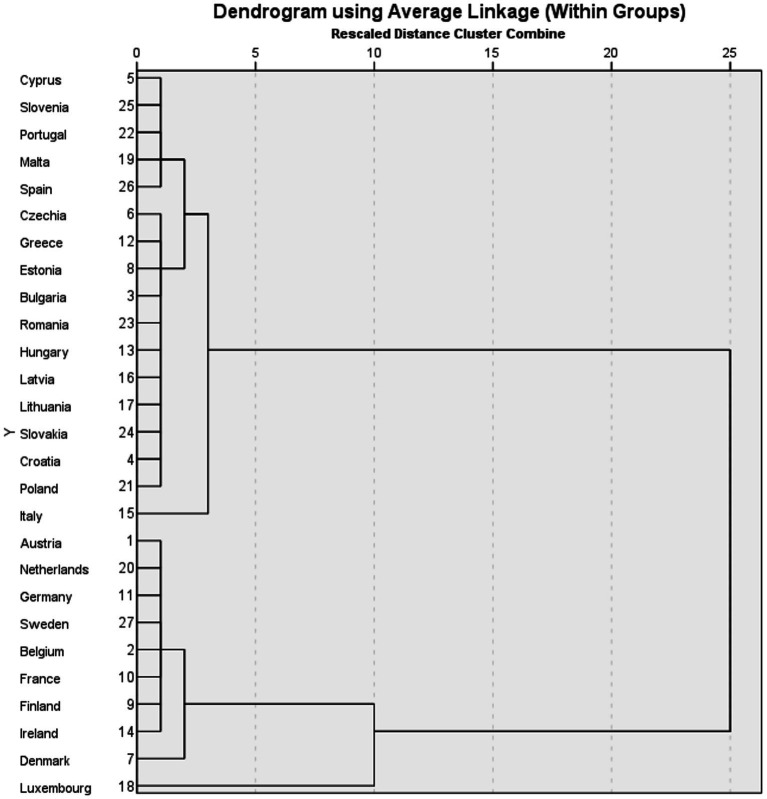
Dendrogram. Source: authors’ design using SPSS v.27.

To explore hypothesis H2, we conducted cluster analysis using the method of within-groups average linkage with a squared Euclidean distance interval. The resulting dendrogram, depicted in Figure 3, illustrates the clustering of countries into homogeneous groups.

The first cluster comprises countries characterized by high values across the selected research variables (Table 5). Notably, Ireland stands out with the highest percentage of individuals reporting good or excellent health status, at 83.7%, followed by several other countries such as Austria, Netherlands, Sweden, and Belgium, all with percentages exceeding 70%. Additionally, Sweden leads in the number of healthy years for individuals aged 65, with 15.9 years, followed closely by Ireland and Germany. In terms of healthcare expenditures, Luxembourg tops the list with total expenditures of 5,875.34 euros per inhabitant, followed by Denmark, the Netherlands, Ireland, and Germany. Surprisingly, countries with the highest healthcare expenditures, such as Luxembourg, Denmark, and the Netherlands, do not necessarily have the highest percentages of individuals reporting good health, indicating a lack of direct correlation between healthcare spending and health outcomes. However, there appears to be a positive association between higher social protection and healthcare expenditures and healthier populations, as reflected in higher GDP *per capita*.

**Table 5:** Cluster 1.

	HS1	HS2	HE1	HE2	SPE1	SPE2
Austria	74.0	8.1	4865.28	11.39	14511.96	29.97
Netherlands	77.9	9.9	5108.39	11.14	14973.43	23.94
Germany	63.9	11.1	5192.41	12.82	13509.79	28.74
Sweden	76.5	15.9	5260.21	11.33	13597.06	25.46
Belgium	75.4	10.8	4462.34	11.20	13021.27	28.98
France	68.4	11.1	4159.55	12.16	13034.78	33.09
Finland	70.0	9.9	4137.71	9.61	13724.60	27.89
Ireland	83.7	11.9	5311.33	7.10	11606.48	14.16
Denmark	71.3	11.2	5642.26	10.53	17585.71	26.82
Luxembourg	73.6	10.9	5875.34	5.77	24823.13	21.49
Cluster means	73.5	11.1	5001.5	10.3	15038.8	26.1
UE mean	68.1	9.0	2858.4	9.2	8310.3	23.2

Source: authors’ design using SPSS v.27.

On the other hand, the second cluster comprises countries with lower values across the selected research variables (Table 6). For instance, Lithuania reports the lowest percentage of individuals with a good or excellent perception of health, at 44.3%, compared to Greece with 78.6%. The number of healthy years at age 65 is also lower in this cluster, with a mean of 7.8 years compared to the EU average of 9.0 years. Moreover, total healthcare expenditures in this cluster are significantly lower than the EU average, ranging from 712.57 euros per inhabitant in Romania to 2689.82 euros per inhabitant in Italy. Similarly, social protection expenditures are lower in this cluster compared to the EU mean, with Croatia reporting the lowest value and Italy the highest.

**Table 6:** Cluster 2.

	HS1	HS2	HE1	HE2	SPE1	SPE2
Cyprus	77.5	7.3	2063.70	8.41	5923.02	22.48
Slovenia	67.2	10.3	2109.68	9.45	5824.37	25.11
Portugal	51.3	7.7	2049.89	10.55	5354.66	24.62
Malta	75.7	12.8	2747.22	10.84	5090.18	19.25
Spain	73.0	11.6	2537.76	10.71	7089.21	27.57
Czechia	63.4	7.5	1859.16	9.24	4430.58	21.28
Greece	78.6	7.6	1469.31	9.51	4547.81	26.53
Estonia	58.4	7.1	1564.65	7.75	3969.42	18.27
Bulgaria	66.7	9.3	753.65	8.52	1661.23	18.18
Romania	73.0	5.9	712.57	6.27	2027.42	16.83
Hungary	62.1	7.6	1031.55	7.30	2588.09	17.67
Latvia	49.7	4.4	1154.22	7.45	2766.14	16.49
Lithuania	44.3	5.9	1335.32	7.54	3472.47	18.62
Slovakia	65.3	4.7	1219.91	7.23	3352.20	19.04
Croatia	63.7	5.0	962.91	7.77	3008.81	23.34
Poland	61.6	8.2	901.94	6.49	3292.14	21.16
Italy	73.3	10.5	2689.82	9.63	9591.40	28.76
Cluster means	65.0	7.8	1597.8	8.5	4352.3	21.5
UE mean	68.1	9.0	2858.4	9.2	8310.3	23.2

Source: authors’ design using SPSS v.27.

Overall, the clustering analysis reveals substantial disparities among EU countries in terms of population health status, healthcare expenditures, and social protection levels. While each country faces challenges in providing sustainable healthcare and social protection systems, those investing more in these sectors tend to have better population health outcomes. In conclusion, hypothesis H2 is supported, as EU countries can indeed be grouped into homogeneous clusters based on health status, healthcare expenditure, and social protection expenditure.

## Discussion

The paper’s findings are broadly consistent with prior studies that emphasize the solid link between social protection and public health within the European Union. Like the previous studies cited (47, 50), our research also suggests that improving well-being through social protection measures can positively impact the quality of life and overall health of EU citizens. This alignment underscores the importance of understanding and leveraging these connections to inform the development of policies aimed at promoting a healthier and more equitable society within the EU. Health investments are essential for the European Union in addressing the challenges identified in its health strategy, especially considering the accentuated impact of the economic crisis. The EU faces critical factors, such as an increasing older adult population, a rising number of chronic diseases, higher demands for medical assistance, and elevated costs associated with technological advancements in healthcare (35). Achieving better value for money through appropriate reforms and investments in the healthcare system is crucial to addressing these challenges (54).

Health investments can bring significant benefits by improving the efficiency and quality of medical services without necessarily incurring higher costs (55). Through appropriate reforms and efficient resource management, more thoughtful use of healthcare funds can lead to savings and better health outcomes for the population (43).

In this paper, we aimed to investigate a hypothesis (H1) suggesting that social protection expenditure positively influences health status in EU countries, and this influence occurs through healthcare expenditure. To test this hypothesis, we employed structural equation modeling to analyze the relationships between variables and to examine whether healthcare expenses mediate the effect of social protection expenditures on health status. The results obtained from structural equation modeling support hypothesis H1. According to the research findings, we identified a significant positive correlation between healthcare expenditures and health status, confirming previous research conclusions (1, 28, 43, 56, 57) about the importance of healthcare investments in promoting a healthy population and societal well-being. We found a significant positive influence of social protection expenditures on health status mediated through healthcare expenditure. A greater allocation of resources towards social protection is frequently related to larger vulnerable groups with meager health. This fact explains the negative influence of social protection expenditures on health status in EU countries. Directing social protection expenditures towards health-related aspects improves the population’s health in EU countries and consequently reduces the future demand for social protection due to fewer vulnerable population groups. A higher allocation of resources in social protection can facilitate access to quality medical services, thereby promoting better population health. These results suggest that an integrated and balanced approach combining social protection and healthcare expenditures can improve health and well-being in the EU population.

Hypothesis H2 suggests that EU countries can be grouped into homogeneous clusters based on health status, healthcare expenditures, and social protection expenditures. To test this hypothesis, we used cluster analysis to examine relevant data from a set of European countries. The results obtained from cluster analysis largely confirm hypothesis H2. We identified distinct groups of countries with similar characteristics regarding health status, healthcare expenditures, and social protection expenditures. These clusters provide relevant insights into the health and well-being situation in various regions of the European Union.

One provoking aspect observed in the cluster analysis is the existence of groups of countries with high levels of healthcare and social protection expenditures but varying health statuses. Within the cluster analysis, we noticed that certain countries stand out due to specific characteristics that position them uniquely in the European context. For example, countries like Luxembourg and Denmark stood out for high healthcare and social protection expenditures, accompanied by relatively good health status. In contrast, countries like Bulgaria and Romania recorded low levels of expenditures, as well as health and social protection.

It is essential to mention that these conclusions are based on the data available during analysis, and the situation may evolve. However, cluster analysis offers a valuable perspective on the diversity and similarities among EU countries regarding healthcare and social protection domains.

Health status affects work capacity, pushing entire groups towards the category of vulnerable populations with low incomes, who access social protection services more frequently (58). Investing in social protection services alone is insufficient to protect populations from poverty. The paper advocates for effective investments in healthcare and social protection systems to combat poverty and social exclusion. Improving the population’s health level strengthens employability, drawing more individuals out of vulnerable segments and breaking the vicious cycle of poor health, poverty, and social exclusion (43).

Regarding social security policy, the EU complements the activities of member states by encouraging cooperation and sharing best practices (27). It recognizes the importance of adequate social protection for vulnerable groups and self-employed individuals in atypical work conditions. Cross-border workers and greater labor mobility require consolidated cooperation to prevent unfair social competition and ensure fair working conditions. The EU’s focus on health and social policies aims to promote a healthier and more inclusive society while recognizing the diversity and autonomy of the national social security system (27).

Consistent with prior studies, our research highlights the importance of social protection measures in enhancing the well-being and overall health of EU citizens. Furthermore, the study supports the assertion that health investments are essential for addressing the challenges outlined in the EU’s health strategy, particularly considering the impact of economic crises and demographic shifts.

### Theoretical implications

By examining various aspects of social protection, this study aims to highlight how these policies can improve health outcomes and the overall well-being of EU citizens. Adequate social protection can provide financial support and assistance to individuals, enabling them to afford essential medical services and treatments. This fact could lead to earlier detection and better management of diseases, as well as reduce health disparities among different socio-economic groups.

Social protection policies can act as a buffer against economic instability and social determinants of health. Unexpected economic events can negatively impact health, as individuals facing financial stress may neglect their well-being. Social protection programs, such as unemployment benefits or housing support, can provide a safety net during difficult times, reducing the potential negative impact on health outcomes. By targeting vulnerable populations and providing adequate resources, social protection measures can contribute to narrowing health disparities among different social categories, fostering a more inclusive and healthier society overall.

The paper emphasizes the importance of robust social protection policies in promoting better health outcomes and improving the well-being of individuals in the EU. By understanding these potential connections, decision-makers can work towards developing more effective social protection strategies that positively impact public health throughout the region.

However, special attention must be given to understanding the complexity and specificities of each national healthcare system. Solutions are not universally applicable and must be tailored to each country’s needs and population. In this regard, the exchange of best practices and international collaboration between health economists and decision-makers can be a valuable tool to identify the best approaches and policies to ensure sustainable and efficient healthcare services for all citizens.

### Practical and managerial implications

Through this study, decision-makers have the potential to improve specific policies and interventions to ensure better health and well-being for EU citizens. Social and healthcare policies and programs can be adapted to provide specific support to vulnerable groups, improving access to medical and social services and reducing health disparities among different population strata. From a managerial perspective, the paper offers valuable information for the optimal allocation of financial and human resources. Identifying the connections between social protection and adopting healthy behaviors can guide the development of interventions to encourage healthy habits and prevent disease onset. The paper serves as an essential source of information for decision-makers in the social and medical domains. It provides a complex perspective on the interactions between social protection and health for EU citizens.

This comprehensive understanding can catalyze policy changes that prioritize the most vulnerable populations, ensuring their access to essential services and resources. By integrating these findings into policy formulation, decision-makers can foster a more inclusive and equitable society where health outcomes are not predetermined by socio-economic status or other factors. Public health practitioners can leverage these insights to design targeted interventions that address the specific needs of marginalized communities, thereby fostering healthier behaviors and reducing the burden of preventable diseases. Moreover, by aligning social protection mechanisms with health promotion efforts, policymakers can create synergies that amplify the impact of interventions and promote sustainable improvements in population health. In essence, this research provides a roadmap for policymakers and practitioners to collaborate effectively in addressing the complex interplay between social factors and health outcomes, ultimately leading to a healthier and more resilient EU population.

### Limitations and future research

Although the study contributes to understanding the relationships between social protection and population health in the EU, it also presents certain limitations. A significant limitation may be connected to measuring social protection and health. Both notions are multifaceted and can be influenced by many factors, including socio-economic and cultural settings. Thus, describing and adequately quantifying these variables can pose methodological challenges.

Longitudinal studies could provide a more comprehensive perspective on the evolution of this relationship over time. Additionally, future research could explore and analyze in more detail the intermediary or mediating factors underlying the connections between social protection and health. Understanding the precise mechanisms through which social protection influences health can provide valuable information for developing appropriate policies and interventions.

Moreover, it is essential to mention that the specific context of the European Union and other regional particularities can influence the data and results obtained in this study. Therefore, it is necessary to continue research and analysis in this field to gain a more complex and detailed perspective on the relationships between social protection expenditures, healthcare expenditures, and health status in EU countries.

## Conclusion

The study presents compelling evidence of the significant association between social protection measures and improved health outcomes among EU member states. Notably, higher levels of social protection expenditure correlate with decreased mortality rates, increased life expectancy, and improved access to healthcare resources, emphasizing the crucial role of robust social protection programs in promoting better health among EU residents.

Utilizing structural equation modeling (SEM) provides a sophisticated approach to exploring the intricate relationship between social protection initiatives and health status while considering potential confounding factors. This methodological advancement enhances the understanding of how social protection influences health outcomes in the EU, offering valuable insights for policy formulation and intervention design.

The research underscores the imperative of prioritizing and investing in social protection policies to enhance overall public health in the European Union. Cohesive and well-designed social protection measures are vital for improving individual health, fostering social cohesion, and reducing health disparities across diverse population segments. Strategic resource allocation and policy alignment with citizens’ health needs are essential for fostering a healthier and more equitable society.

Future research should continue to explore various social, economic, and cultural factors influencing this dynamic interaction, recognizing the complexity of the relationship between social protection and health status. As the EU faces ongoing health challenges and societal risks, sustained efforts to design and implement inclusive social protection measures are crucial for fostering healthier and more prosperous communities throughout the region. Embracing evidence-based policy interventions is paramount for the EU to reaffirm its commitment to enhancing the health and quality of life of its diverse population. Moreover, further research is needed to delve deeper into understanding how social protection contributes to better health outcomes, considering additional factors such as socio-economic determinants and cultural influences.

## Data availability statement

The original contributions presented in the study are included in the article/supplementary material, further inquiries can be directed to the corresponding author.

## Ethics statement

This study did not require ethical review and approval following the local legislation and institutional requirements.

## Author contributions

CB: Conceptualization, Data curation, Formal analysis, Investigation, Methodology, Project administration, Resources, Software, Supervision, Validation, Visualization, Writing – original draft, Writing – review & editing. AV: Conceptualization, Data curation, Formal analysis, Investigation, Methodology, Project administration, Resources, Software, Supervision, Validation, Visualization, Writing – original draft, Writing – review & editing.
